# Adaptive Radiation in Mediterranean *Cistus* (Cistaceae)

**DOI:** 10.1371/journal.pone.0006362

**Published:** 2009-07-23

**Authors:** Beatriz Guzmán, María Dolores Lledó, Pablo Vargas

**Affiliations:** 1 Real Jardín Botánico - CSIC, Madrid, Spain; 2 Royal Botanic Gardens, Kew, Richmond, Surrey, United Kingdom; American Museum of Natural History, United States of America

## Abstract

**Background:**

Adaptive radiation in Mediterranean plants is poorly understood. The white-flowered *Cistus* lineage consists of 12 species primarily distributed in Mediterranean habitats and is herein subject to analysis.

**Methodology/Principal Findings:**

We conducted a “total evidence” analysis combining nuclear (ncp*GS*, ITS) and plastid (*trnL-trnF*, *trnK-matK*, *trnS-trnG*, *rbcL*) DNA sequences and using MP and BI to test the hypothesis of radiation as suggested by previous phylogenetic results. One of the five well-supported lineages of the *Cistus-Halimium* complex, the white-flowered *Cistus* lineage, comprises the higher number of species (12) and is monophyletic. Molecular dating estimates a Mid Pleistocene (1.04±0.25 Ma) diversification of the white-flowered lineage into two groups (*C. clusii* and *C. salviifolius* lineages), which display asymmetric characteristics: number of species (2 vs. 10), leaf morphologies (linear vs. linear to ovate), floral characteristics (small, three-sepalled vs. small to large, three- or five-sepalled flowers) and ecological attributes (low-land vs. low-land to mountain environments). A positive phenotype-environment correlation has been detected by historical reconstructions of morphological traits (leaf shape, leaf labdanum content and leaf pubescence). Ecological evidence indicates that modifications of leaf shape and size, coupled with differences in labdanum secretion and pubescence density, appear to be related to success of new species in different Mediterranean habitats.

**Conclusions/Significance:**

The observation that radiation in the *Cistus salviifolius* lineage has been accompanied by the emergence of divergent leaf traits (such as shape, pubescence and labdanum secretion) in different environments suggets that radiation in the group has been adaptive. Here we argued that the diverse ecological conditions of Mediterranean habitats played a key role in directing the evolution of alternative leaf strategies in this plant group. Key innovation of morphological characteristics is supported by our dated phylogeny, in which a Mediterranean climate establishment (2.8 Ma) predated the adaptive radiation of the white-flowered *Cistus*.

## Introduction

The concept of adaptive radiation implies a rapid ecological diversification, which should be reflected in a greater morphological and/or physiological divergence among species in brief periods of rapid diversification from a single ancestor [1], [2]. Two mechanisms could generate adaptive radiations: (1) extrinsic causes due to new environmental circumstances [3], [4]; (2) intrinsic characters of organisms (key innovation) that allow a taxon to utilize existing niche space in a novel manner [5]. Remoteness and the rich diversity of habitats of island systems help ensure little competition and different environments to test the potential of plant radiations [6], [7]. In contrast to the wealth of studies documenting adaptive radiations in oceanic islands [see 3], [8], [9] and particular mainland habitats [see 10], [11], we have found in literature no study fully focused on the Mediterranean region.

The Mediterranean climatic type, characterized by a strong seasonality (hot dry summers, cool wet winters), occurs in California, South Africa, central Chile, southern Australia, and typically in the Mediterranean Basin [12], [13]. In all five of these areas the native vegetation is a dense scrub characterized by annuals, drought-tolerant deciduous and semi-deciduous malacophyllous species, and woody evergreen sclerophyllous species [14]. Sclerophyllous species are adapted to low water availability during summer by means of small, leathery and dark leaves covered with thick cuticles and small, thick-walled cells [15]. Small leaves and low specific leaf area have been viewed as adaptations to Mediterranean-type climates in many species of evergreen plants [16]. Indeed, sclerophylly is so successful that unrelated genera and families of woody plants converged into similar leaf traits. Two alternative origins have been proposed for the evolution of Mediterranean, woody plants: resprouters corresponding to older lineages (Tertiary with tropical to subtropical conditions) and seeders (such as *Cistus*) to younger lineages (Quaternary with Mediterranean conditions) [17]. Few studies have, however, addressed the origin of Mediterranean plant groups by means of phylogenetic approaches related to ecological preferences [but see 18].

**Figure 1:**
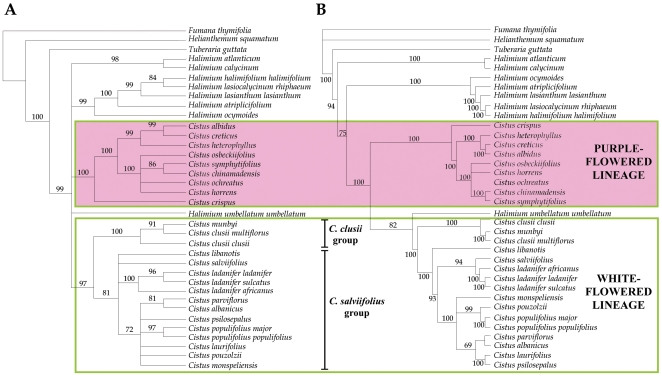
Phylogenetic hypothesis based on plastid (*trnL-F*, *trnK-matK*, *trnS-trnG*, *rbcL)* and nuclear (ITS, ncp*GS)* sequences. (A) Strict consensus of 104 equally parsimonious trees of 1317 steps (CI = 0.82, RI = 0.80), showing bootstrap support for clades above branches; (B) Bayesian inference tree (50% majority rule consensus tree) showing posterior probabilities above branches.

Significant shrub components in the European-African Mediterranean ecosystems (e.g., “maquis”, “garrigue”) belong to Cistaceae (*Tuberaria*, *Halimium*, *Cistus*). *Cistus* is a genus of 21 frutescent and suffrutescent shrub species with a predominantly Mediterranean distribution [19], except for five species endemic to the Canary Islands (Table 1). Previous phylogenetic studies revealed the separation of the *Cistus*-*Halimium* lineage and identification of two major natural groups: one of purple-flowered *Cistus* species (hereafter the purple-flowered lineage) and other containing the white-flowered species of *Cistus*, plus the pinkish-flowered *C. parviflorus* (hereafter the white-flowered lineage) [20], [21], [22]. Moreover, the white-flowered lineage is divided in two groups: one containing *C. clusii* and *C. munbyi* species (hereafter the *C. clusii* group) and other containing the rest of the white-flowered *Cistus* species (9), plus *C. parviflorus* (hereafter the *C. salviifolius* group) (Fig. 1). Despite the two lineages (the *C. clussi* and the *C. salviifolius* groups) are inhabiting the Mediterranean basin, the *C. salviifolius* group has undergone higher differentiation and displays greater variation in leaf trichome density, size, shape and tissue thickness than do the *C. clusii* group. These properties influence the resistance to drought stress and solar irradiance [14]. Indeed, ecological analyses of leaf morphological and physiological characters in dry environments [23], [24] appear to be related to speciation of Mediterranean plants.

**Table 1:** List of species used in the phylogenetic analysis.

*Taxon*	*Distribution*	*Locality/source*	*Voucher*
*Cistus* L.			
*Cistus albanicus* E.F. Warb. ex Heywood	Albania, Greece	Cultivated	R. G. Page 8cBGA04 (MA)
*Cistus albidus* L	Iberia, S France, N Italy, N Africa, Corsica, Sardigna	Spain, Madrid, Aldea del Fresno	P. Vargas 25PV03 (MA)
*Cistus chinamadensis* Bañares et Romero	La Gomera, Tenerife (Canary Islands)	Canary Islands, La Gomera	Á. Fernández & J. Leralta 44BGA04 (MA)
			R. G. Page 8bBGA04 (MA)
*Cistus clusii* Dunal subsp. *clusii*	Spain, Italia, N Africa, Sicily	Spain, Málaga, Mijas	C. Navarro *et al.* (MA618671)
*Cistus clusii* Dunal subsp. *multiflorus* Demoly	Balear Islands, SE Iberia Peninsula	Spain, Balear Islands, Mallorca, Sa Rápita	P. Vargas 209PV04 (MA)
*Cistus creticus* L.	Mediterranean Basin	Greece, Olympus	B. Guzmán 58BGA04 (MA)
*Cistus crispus* L.	Iberia, S France, N Italy, N Africa, Corsica, Sicily	Spain, Córdoba, Posadas	B. Guzmán 99BGA04 (MA)
*Cistus heterophyllus* Desf.	SE Spain, N Africa	Morocco, Beni-Hadifa	B.Guzmán 2BGA05 (MA)
*Cistus horrens* Demoly	Gran Canaria (Canary Islands)	Canary Islands, Gran Canaria, Ayacata	B. Guzmán 109BGA04 (MA)
*Cistus ladanifer* L. subsp. *africanus*	S Spain, N Africa	Morocco, Targuist	B. Guzmán 7BGA03 (MA)
*Cistus ladanifer* L. subsp. *ladanifer*	S France, Iberia, N Africa, Cyprus	Spain, Madrid, Boadilla del Monte	B. Guzmán 29BGA04 (MA)
*Cistus ladanifer* L. subsp. *sulcatus*	S Portugal	Portugal, Sagres	B. Guzmán 13BGA03 (MA)
*Cistus laurifolius* L.	N Africa, Iberia, France, Italy, Corsica, Turkey	Spain, Jaén, Sierra de Segura	R. G. Page 149BGA04 (MA)
*Cistus libanotis* L.	Portugal, S Spain, Argelia	Spain, Córdoba	B. Guzmán 35BGA04 (MA)
*Cistus monspeliensis* L.	Mediterranean Basin, Canary Islands	Portugal, Sagres	O. Filippi 4BGA04 (MA)
*Cistus munbyi* Pomel	Algeria, Morocco	Morocco	R. G. Page 8BGA04 (MA)
*Cistus ochreatus* C. Sm. ex Buch	Gran Canaria (Canary Islands)	Canary Islands, Gran Canaria	P. Escobar 48/05 (MA)
*Cistus osbeckiifolius* Webb ex Christ	Tenerife (Canary Islands)	Canary Islands, Tenerife	O. Filippi 6BGA04 (MA)
*Cistus parviflorus* Lam.	Greece, Turkey, Italy, Cyprus, N Libia, Lampedusa	Greece, Crete	B. Guzmán 20BGA04 (MA)
*Cistus populifolius* L. subsp. *major* (Dunal) Heywood	Iberia, N Morocco	Portugal, Ourique	P. Vargas 5PV03 (MA)
*Cistus populifolius* L. subsp. *populifolius*	Iberia, S France	Spain, Ávila, Arenas de San Pedro	R. G. Page 8tBGA04 (MA)
*Cistus pouzolzii* Delile	Algeria, N Morocco, France	France	P. Vargas 7PV03 (MA)
*Cistus psilosepalus* Sweet	Iberia, France	Spain, Ávila, Arenas de San Pedro	P. Vargas 6PV03 (MA)
*Cistus salviifolius* L.	Mediterranean Basin	Spain, Ávila, Arenas de San Pedro	B. Guzmán 143BGA04 (MA)
*Cistus symphytifolius* Lam.	El Hierro,La Palma,La Gomera,Tenerife,Gran Canaria	Canary Islands, La Palma, La Cumbrecita	
*Fumana* (Dunal) Spach			
*Fumana thymifolia* (L.) Spach ex Webb	Mediterranean Basin	Portugal, Ferrerías	B. Guzmán 53BGA04 (MA)
Halimium (Dunal) Spach			
*Halimium atlanticum* Humbert & Maire	N Africa	Morocco, Tazzeka	RDG14/2006/5
*Halimium atriplicifolium* (Lam.) Spach	Spain, N Morocco	Spain, Granada, Sierra Nevada	P. Vargas 120PV04 (MA)
*Halimium calycinum* (L.) K. Koch	Iberia, NW Morocco	Portugal, Cabo Sardao	B. Guzmán 49BGA04 (MA)
*Halimium halimifolium* (L.) Willk. *halimifolium*	Iberia, Morocco	Spain, Málaga, Marbella	A. Segura (MA580185)
*Halimium lasianthum* (Lam.) Spach *lasianthum*	SW Iberia, N Morocco	Spain, Málaga	P. Vargas 3PV06
*Halimium lasiocalicynum* (Boiss. & Reut.) Gross ex Engl. subsp. *riphaeum* (Pau & Font Quer) Maire	N Africa	Morocco, Bab-Berred	P. Escobar 665/04 (MA)
*Halmium ocymoides* (Lam.) Willk.	Iberia Peninsula, N Morocco	Portugal, Coimbra	R. G. Page 158BGA04 (MA)
*Halimium umbellatum* (L.) Spach	Mediterranean Basin	Spain, Madrid, Tres Cantos	P. Vargas 71BGA04 (MA)
*Helianthemum* Mill.			
*Helianthemum squamatum* (L.) Dum. Cours.	Iberia, N Africa	Cultivated	B. Guzmán 70BGA04 (MA)
*Tuberaria* Dunal			
*Tuberaria guttata* (L.) Fourr.	W Europe, Mediterranean Basin, Canary Islands	Portugal, Vila do Vispo	B. Guzmán 44BGA04 (MA)

In this study, we used a molecular phylogenetic approach of DNA sequence data, sampled from both the nuclear (ITS, ncp*GS*) and the plastid (*trnL-trnF*, *trnK-matK*, *trnS-trnG*, *rbcL*) genomes, to test the explicit hypothesis of adaptive radiation. We first explored single ancestry in the *Cistus*-*Halimium* complex and differentiation in short periods of time by means of phylogenetic and molecular clock analyses [1], [25]. To test evolution in Mediterranean conditions, we chose a lineage exclusive to the Mediterranean basin (*C. salviifolius* lineage). Phenotype-environment correlation was further conducted to infer the role of ecological and vegetative characteristics [26], [27], [28] involved in speciation of this group.

## Results

### Phylogenetic analyses

The characteristics of the six sequence data sets are summarized in Table 2. MP analysis using Fitch parsimony resulted in 104 shortest trees of length 1317 steps (Fig. 1) for the combined sequence matrix. The consistency index (CI) for these trees was 0.82 and the retention index (RI) was 0.80. The BI tree displayed similar topology (except for the *Halimium umbellatum* position) and support values. Plastid and nuclear datasets yielded a similar phylogenetic pattern, although plastid sequences provided a more resolved tree (results not shown). In addition to strong (99% BS, 94 PP) support for the monophyly of the *Cistus-Halimium* complex, parsimony and Bayesian consensus trees were consistent at different places: (1) *Cistus* species were not monophyletic; (2) *Cistus* species were divided in two lineages, one of purple-flowered species (except *C. parviflorus*) (100% BS, 100 PP) and other of white-flowered species plus *C. parviflorus* (97% BS, 100 PP); (3) *Cistus crispus* was the sister-group of the rest of purple-flowered species (100% BS, 100 PP); and (4) a sister-group relationship existed between the *C. clusii* group (100% BS, 100 PP) and the rest of the white-flowered species plus *C. parvifloru*s (81% BS, 100 PP). *Halimium umbellatum* appears to be related to the white-flowered lineage in the Bayesian analysis, but not in the MP analysis (Fig. 1).

**Table 2:** Characteristics of each of the DNA sequence regions used in the phylogenetic analysis of Cistaceae and the white-flowered *Cistus*.

	*trnS-trnG*	*trnL-trnF*	*trnK-matK*	*rbcL*	ITS	ncp*GS*
**Cistaceae**						
** Length (bp)**						
** Total aligned length**	1084	516	1403	1404	697	402
** Length range - ingroup**	617–824	399–461	1302–1357	1403–1404	644–650	340–452
** Length range - outgroup**	158–684	377–422	1301–1316	1404	585–654	318
** Number of characters**						
** Total included**	713	516	1403	1379	697	402
** Variable/parsimony-informative**	148/54	128/52	280/108	103/44	203–69	86/17
** Mean G+C content**	21%	33%	33%	43%	65%	40%
** Maximum sequence divergence (GTR)**	17.92%	14.1%	14.08%	4.11%	20.37%	35.4%
** Sequence evolution model (Akaike Test)**	GTR+G	GTR+G	GTR+G	GTR+I	GTR+I+G	HKY+G
**White-flowered ** ***Cistus*** ** plus ** ***C. parviflorus***						
** No. of variable/parsimony-informative characters**	45/25	28/11	33/12	20/10	75/33	25/8
** Maximum sequence divergence (GTR)**	1.90%	3.15%	0.85%	0.74%	4.21%	3.11%
** Sequence evolution model (Akaike Test)**	GTR+I	F81+I	GTR	HKY	HKY+I+G	HKY+G

### Evaluating patterns of trait evolution

**Figure 2:**
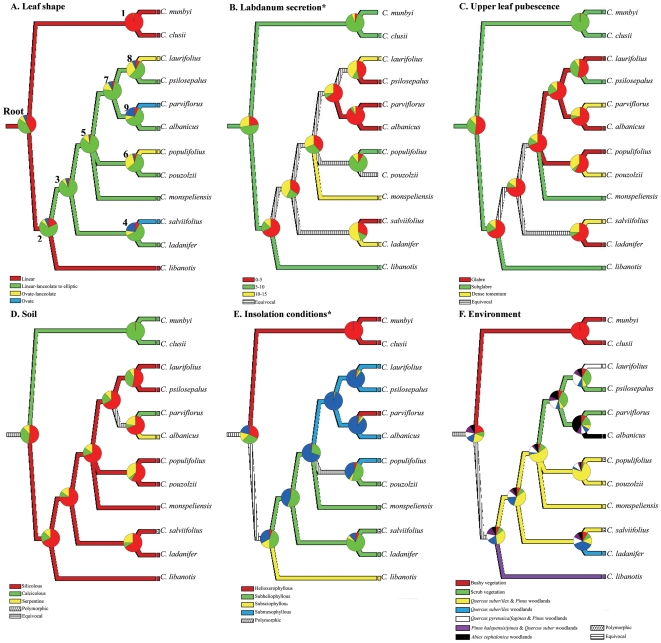
Historical patterns of leaf characters and ecological attributes. Key characteristics are mapped onto the MacClade optimization tree as inferred by the Bayesian analysis: (A) Leaf shape, (B) Leaf labdanum secretion [49], (C) Pubescence of upper leaf surface, (D) Soil requirements, (E) Insolation conditions [77], (F) Environment. Pie charts at nodes represent the posterior probabilities of Bayesian inference character state evolution (Table 2). Node coding above branches in Fig. 2A.

The range of interspecific variation in leaf morphology and ecological requirements is shown in Table 3. Character reconstruction of three morphological and three ecological characters mapped on the Bayesian consensus tree (Fig. 2) using MacClade optimization and Bayesian inference to investigate patterns of evolution. The most relevant results from the historical reconstructions are following described:

1. **Leaf shape (** **Fig. 2A** **).** The character state reconstruction showed linear or linear-lanceolate to elliptic leaves as a plesiomorphic state. Ovate-lanceolate and ovate shapes evolved twice in the *C. salviifolius* lineage.
2. **Labdanum secretion (** **Fig. 2B** **).** The character was equivocal in most of the *C. salviifolius* lineage because, in part, of missing data from two species (*C. mumbyi*, *C. pouzolzii*). A medium percentage (5–10%) of secretion per unit leaf dry weight was, however, traced as the most likely ancestral state.
3. **Upper leaf pubescence (** **Fig. 2C** **).** The character was revealed as very homoplastic within the *C. salviifolius* lineage. Despite the reconstruction was equivocal tracing the state at some nodes, independent acquisition (up to three times) of a dense tomentum is interpreted. Shifts between glabrous and subglabrous leaves appeared dynamic.
4. **Soil (** **Fig. 2D** **).** The historical reconstruction traced silicolous soils as the ancestral state for the *C. salviifolius* lineage. It was noteworthy that the only two species inhabiting basic (*C. parviflorus*) and ultrabasic (*C. albanicus*) soils within this lineage are sister species.
5. **Insolation conditions (** **Fig. 2E** **).** Character optimization was equivocal reconstructing the ancestral state in the *C. salviifolius* lineage. Two sister species groups underwent a dramatic change in insolation conditions (*C. parviflorus*-*C. albanicus*; *C. populifolius*-*C. pouzolzii*). Although ancestral character states were poorly optimised for insolation conditions, reversal to high solar exposure (helioxerophyllous) was unequivocally acquired for *C. parviflorus*.
6. **Environment (** **Fig. 2F** **).** A high frequency in habitat change was found in the *C. salviifolius* lineage. Similar environments were shared in a few groups with (*C. pouzolzii, C. populifolius*) or without (*C. psilosepalus*, *C. parviflorus*) sister relationships. In contrast, three habitats were occupied by four closely-related species (*C. laurifolius*, *C. psilosepalus*, *C. parviflorus*, *C. albanicus*) suggesting a dynamic habitat change in the course of evolution of the *C. salviifolius* lineage.

**Table 3:** Morphological and environmental characteristics of the white-flowered *Cistus* lineage. Data were taken from Grosser [75], Martín & Guinea [76], Dansereau [77] ^*^, Warburg [78], Demoly and Montserrat [79], Greuter [80], Gülz *et al*. [49] ^**^ and own observations.

	Soil	Climate conditions	Altitude (m)	Insolation conditions^*^, environment	Leaf shape (length×width in mm)
*C. albanicus*	serpentines	mesic, Mediterranean mountain	1000–1500	submesophyllous, *Abies cephalonica* woodlands	elliptic (3–5×0.8–1.5)^1^
*C. clusii*	calcicolous	dry to semi-arid, Mediterranean coast	0–1500	helioxerophyllous, bushy vegetation	linear (10–26×1–2)
*C. ladanifer*	silicolous	dry, Mediterranean	(0) 300–1000 (1500)	subheliophyllous, degradated *Quercus suber/ilex* woodlands	linear-lanceolate (40–80×6–21)
*C. laurifolius*	silicolous	mesic, Mediterranean mountain	1900 (400–2800)	submesophyllous, degradated *Q. pyrenaica/faginea* and *Pinus* woodland	ovate-lanceolate (40–90×17–30)
*C. libanotis*	silicolous, sandy	dry, Mediterranean coast	0–500 (1200)	subsciophyllous, degradated *Pinus halepensis/pinea* and *Quercus suber* woodlands	linear (22–40×2–5)
*C. monspeliensis*	silicolous	dry, Mediterranean	0–800 (1200)	subheliophyllous, degradated *Quercus suber/ilex* and *Pinus* woodlands	linear-lanceolate (15–45×2–7)
*C. munbyi*	calcicolous	Mediterranean coast	0–100	helioxerophyllous, bushy vegetation	linear (6–30×1–4)
*C. parviflorus*	calcicolous	dry, Mediterranean coast	0–600	helioxerophyllous, scrub vegetation	ovate (15–30×7–27)
*C. populifolius*	silicolous	dry, Mediterranean	200–1500	submesophyllous, degradated *Quercus* and *Pinus* woodlands	ovate-lanceolate (50–95×25–55)
*C. pouzolzii*	silicolous	dry, mountain Mediterranean	800–1800	subheliophyllous, degradated *Quercus suber*/*ilex* and *Pinus* woodlands	lanceolate-elliptic (20–31×4–11)
*C. psilosepalus*	silicolous	humid, woodlands of Atlantic influence	0–800 (1100)	submesophyllous, scrub vegetation	lanceolate-elliptic (30–65×10–23)
*C. salviifolius*	silicolous/calcicolous	humid to dry, Mediterranean and Eurosiberian regions	0–1800	subheliophyllous/submesophyllous, degradated woodlands of many types	ovate (8–18×7–12)

	Leaf margin	Leaf venation	Leaf surface and texture	Labdanum secretion^**,^ 2	Leaf non-secretorial trichomes^**^	
					Upper surface	Lower surface
*C. albanicus*	flat	reticulate	smooth, soft	1.0	long, single, stellate	glabre
*C. clusii*	revolute	uni-nerve	smooth, coriaceous	6.0	subglabre with tuft of single hairs	dense tomentum of stellate
*C. ladanifer*	flat	Pinnate	smooth, coriaceous	12.5	glabre	dense tomentum of stellate
*C. laurifolius*	slightly crispate	Parallel	smooth, coriaceous	13.5	glabre	dense tomentum of single and stellate (deciduous)
*C. libanotis*	revolute	uni-nerve	smooth, coriaceous	6.1	subglabre, stellate	dense tomentum of stellate
*C. monspeliensis*	flat, slightly revolute	Parallel	smooth, coriaceous	10.7	subglabre, stellate	dense tomentum of minute stellate
*C. munbyi*	revolute	uni-nerve	smooth, coriaceous	–	subglabre	dense tomentum of stellate
*C. parviflorus*	flat	Parallel	smooth, coriaceous	1.2	dense tomentum, stellate	dense tomentum of stellate
*C. populifolius*	flat	Pinnate	smooth, coriaceous	5.6	glabre	glabre
*C. pouzolzii*	crispate	Parallel	rough, coriaceous	–	dense tomentum, single and stellate	dense tomentum of single and stellate
*C. psilosepalus*	flat	reticulate	smooth, soft	2.0	subglabre, stellate	stellate
*C. salviifolius*	slightly crispate	Pinnate	rough, coriaceous	0.5	stellate	stellate

Note: ^1^ values from 16 leaves.

2 % per unit leaf dry weight.

The BayesTraits analysis of trait evolution was used to test reconstruction uncertainty. Table S2 reports ratedev settings and mean values (±95% confidence intervals) of the log-likelihood and posterior distributions of the rate of coefficients obtained from the reversible jump (RJ) MCMC analysis. The mean of the Bayesian posterior probabilities of each character state at every node (nine nodes) are provided in Table 4 and Fig. 2. The 95% confidence intervals of the posterior probabilities were all lower than±0.004. The Bayesian results mostly supported the MP (MacClade) optimization. Particular points of disagreement between both analyses were: (1) subglabre leaves at the root of the tree in the MP analysis whereas the Bayesian probability (0.52) was higher for glabre leaves; (2) ancestral states at node 2 were reconstructed as linear and subglabre leaves in the MP analysis, while the Bayesian approach estimated a higher probability for linear-lanceolate (0.68) and glabre leaves (0.68) states to be ancestral; (3) the historical reconstruction using the MP optimization traced *Quercus suber/ilex* and *Pinus* woodlands as the ancestral state at node 4 (*C. ladanifer-C. salviifolius*), while *Quercus suber/ilex* woodlands showed the highest posterior probability (0.38); (4) subheliophyllous condition was ancestral at node 5 in the MP optimization but the submesophyllous condition displayed the highest posterior probability (0.70); (5) scrub vegetation was the ancestral state at node 9 (*C. parviflorus-C. albanicus*) using the MacClade optimization, while *Abies cephalonica* woodlands displayed the highest posterior probability (0.42).

**Table 4:** Mean of posterior probabilities of Bayesian inference character state evolution of successive iterations (9,000,000) by RJ MCMC (see text) for six characters.

	Leaf shape^a^	Labdanum secretion^b^	Leaf pubescence^c^	Soil^d^	Insolation conditions^e^	Environment^f^
Root	**0.42**/0.42/0.08/0.07	0.23/**0.49**/0.28	0.52/**0.36**/0.12	0.77/0.17/0.06	0.32/0.31/0.16/0.21	0.21/0.10/0.22/0.14/0.11/0.13/0.09
Node 1	**0.99**/0.00/0.01/0.00	0.05/**0.89**/0.06	0.01/**0.98**/0.01	0.00/**0.99**/0.01	**0.99**/0.00/0.00/0.01	**0.96**/0.01/0.01/0.01/0.01/0.00/0.00
Node 2	**0.18**/0.68/0.07/0.07	0.30/**0.34**/0.36	0.68/**0.19**/0.13	**0.98**/0.01/0.01	0.05/0.45/0.17/0.33	0.06/0.09/0.33/0.18/0.12/0.14/0.08
Node 3	0.03/**0.86**/0.06/0.05	0.35/0.22/0.43	0.75/0.10/0.15	**0.98**/0.01/0.01	0.02/**0.51**/0.02/0.45	0.06/0.09/**0.40**/0.19/0.12/0.06/0.08
Node 4	0.08**/0.60**/0.09/0.23	0.32/0.14/0.54	0.66/0.09/0.25	**0.89**/0.07/0.04	0.06/**0.76**/0.06/0.12	0.09/0.09/**0.13**/0.38/0.13/0.09/0.09
Node 5	0.02**/0.85**/0.11/0.02	0.38/0.31/0.31	0.70/0.15/0.15	**0.96**/0.02/0.02	0.02/**0.27**/0.01/0.70	0.04/0.11/**0.57**/0.04/0.12/0.04/0.08
Node 6	0.04/**0.60**/0.32/0.04	0.09/0.80/0.11	**0.58**/0.06/0.36	**0.98**/0.01/0.01	0.05/0.49/0.05/0.41	0.03/0.03/**0.74**/0.04/0.10/0.03/0.03
Node 7	0.04/**0.73**/0.15/0.08	0.65/0.09/0.26	**0.67**/0.21/0.12	**0.80**/0.08/0.12	0.01/0.02/0.01/**0.96**	0.07/**0.33**/0.07/0.08/0.18/0.08/0.19
Node 8	0.07/**0.55**/0.31/0.07	0.46/0.12/0.42	**0.53**/0.38/0.09	**0.95**/0.02/0.03	0.03/0.04/0.04/**0.89**	0.08/**0.33**/0.08/0.09/0.25/0.08/0.09
Node 9	0.07/**0.62**/0.08/0.23	**0.90**/0.05/0.05	**0.70**/0.07/0.23	0.11/0.31/0.58	0.03/0.04/0.04/**0.89**	0.08/**0.18**/0.08/0.08/0.08/0.08/0.42

The 95% confidence intervals of the posterior probabilities were all less than±0.004. In bold character state evolution as traced in MacClade optimization (Fig. 2). Particular points of disagreement between Bayesian and the MacClade optimization are underlined. Node codes as in Fig. 2A.

Note: Values in the table reflect estimates based on the averaging over 1000 Bayesian tree.

a Leaf shape: linear/linear-lanceolate to elliptic/ovate-lanceolate/ovate.

b Labdanum secretion: 0–5/5–10/10–15% per unit leaf dry weight.

c Leaf pubescence: glabre/subglabre/dense tomentum.

d Soil: silicolous/calcicolous/serpentin.

e Insolation conditions: helioxerophyllous/subheliophyllous/subsciophyllous/submesophyllous.

f Environment: bush/scrub/*Quercus suber*-*ilex*& *Pinus* woodlands/*Quercus suber*-*ilex* woodlands/*Quercus pyrenaica*-*faginea* & *Pinus* woodlands/*Pinus halepensis-pinea* & *Quercus suber* woodlands/*Abies cephalonica* woodlands.

### Bayesian analysis of correlated evolution

Table 5 shows the log-Bayes factor calculations and significance following the scale of Bayes factor test presented by Kass & Raftery [29]. The evolution of leaf traits was not closely associated with ancestral changes in environment and insolation conditions. There was evidence against a correlated evolution of insolation conditions to leaf shape (log-Bayes factor = −1.5) and labdanum secretion (log-Bayes factor = −1.8). Additionally, barely evidence against correlated evolution for leaf pubescence and environment has been found (log-Bayes factor = −0.1). In contrast, barely correlated evolution was suggested between three pairs of variables: leaf shape/environment (log-Bayes factor = 0.7), labdanum secretion/environment (log-Bayes factor = 0.8) and leaf pubescence/insolation conditions (log-Bayes factor = 0.6). As already discussed elsewhere for organism radiations [30], estimates of barely correlated evolution have a strong evolutionary significance considering short tree branches.

**Table 5:** Calculations for log-Bayes factor tests in favour of a dependent model. In the final column, we followed the Bayes factor test [29] in our interpretation of the log-Bayes factor.

	Log-harmonic mean^a^		log-Bayes factor	Significance
	Dependent model	Independent model		
Leaf shape/environment	−12.72	−13.06	0.7	barely in favour
Leaf shape/insolation	−18.12	−17.36	−1.5	against
Labdanum secretion/environment	−11.99	−12.39	0.8	barely in favour
Labdanum secretion/insolation	−17.23	−16.33	−1.8	against
Leaf pubescence/environment	−11.49	−11.44	−0.1	barely against
Leaf pubescence/insolation	−16.25	−16.58	0.6	barely in favour

Note:^a^ Mean calculated from 9,000,000 iterations values.

### Haplotype analysis of the white-flowered *Cistus* lineage

**Figure 3:**
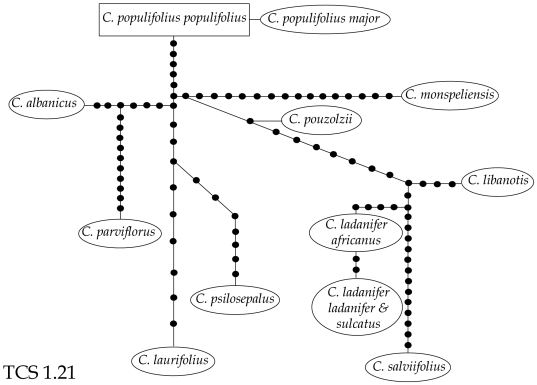
Statistical parsimony network representing relationships of the 12 plastid (*trnL-trnF*, *trnK-matK*, *rbcL*, *trnS-trnG*) haplotypes of the white-flowered *Cistus* lineage. Lines indicate mutation steps (single nucleotide substitutions) and dots (•) represent missing haplotypes (extinct or not found). A star-like shape of the network is congruent with a process of radiation in this group.

Sequence length of the white-flowered *Cistus* lineage was 417–461 bp for *trnL-trnF*, 561–585 for *tnrS-trnG*, 1309–1357 for *trnK-matK* and 1378–1379 for *rbcL* (Table 2). The combined data of plastid sequences for 10 species (13 taxa) of the *C. salviifolius* lineage distinguished only 12 substitution-based haplotypes (Table S3). Haplotypes were exclusive to a single species or subspecies (Table S3), except for one for both *C. ladanifer* subspp. *ladanifer* and *sulcatus*. TCS constructed a single, star-like network (Fig. 3) displaying no loops. This analysis is congruent with a multiple lineage divergence pattern from ancestral haplotypes, as expected in a radiation.

### Estimates of divergence times

**Figure 4:**
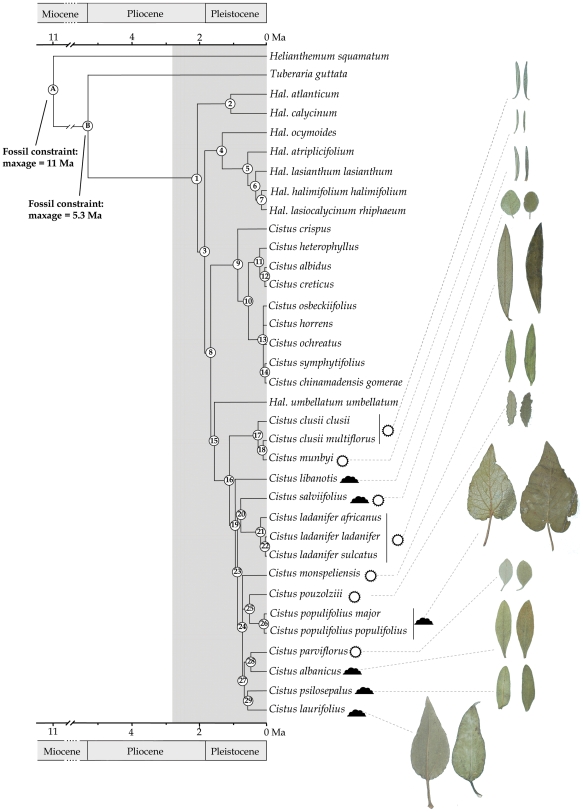
Phylogenetic chronogram of the *Cistus-Halimium* complex based on the Bayesian consensus tree. Fossil calibration points are indicated on the tree. Shaded area delineates the establishment of the Mediterranean climate 2.8 million years ago [50]. Geological timescales are shown both at the top and the bottom. Photographs illustrate diversity in leaf morphology of the white-flowered *Cistus* species (only subsp. *ladanifer* of *C. ladanifer*, subsp. *clusii* of *C. clusii* and subsp. *populifolius* of *C. populifolius* are illustrated). Species insolation conditions [77] are plotted on the right side of the tree (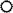, helioxerophyllous and subhelioxerophyllous;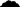, subsciophyllous and submesophyllous).

Results of the dating analysis are shown in Table 6 and Fig. 4. In general, the data indicated a Pliocene-Pleistocene (2.11±0.87 Ma) divergence between the basal-most *Halimium* and *Cistus-Halimium* groups, followed by a Pleistocene differentiation of the major clades of the latter group. An ancestor shared by *Halimium umbellatum* and *Cistus* appeared to have diverged after the Pliocene-Pleistocene boundary (1.47±0.35 Ma). Short branch lengths may reflect a rapid divergence process in the white-flowered lineage. An early divergent lineage of *C. clusii* and *C. mumbyi* (*C. clusii* lineage) at 1.04±0.25 Ma was followed by differentiation of 10 species (*C. salviifolius* lineage) in the Mid Pleistocene (0.88±0.22 Ma). The average per-lineage species diversification rate for the *C. salviifolius* lineage was 1.46–2.44 species per million years.

**Table 6:** Penalized Likelihood (bootstrapping of 100 trees) molecular clock estimates of ages for constrained and unconstrained nodes.

Node	Mean age (Ma)	SD (Ma)	Maximum age (Ma)	Minimum age (Ma)
A (11)	9.65	2.21	11.00	0.58
B (5.3)	4.87	1.10	5.30	0.21
1	2.11	0.87	4.93	0.14
2	1.01	0.31	1.99	0.06
3	1.78	0.45	2.74	0.12
4	1.25	0.32	1.80	0.07
5	0.53	0.15	0.83	0.03
6	0.30	0.09	0.49	0.02
7	0.15	0.06	0.33	0.006
8	1.56	0.38	2.32	0.09
9	0.80	0.21	1.17	0.05
10	0.52	0.14	0.78	0.03
11	0.19	0.07	0.33	0.01
12	0.04	0.02	0.13	0.002
13	0.05	0.06	0.21	0.000
14	0.04	0.02	0.13	0.003
15	1.47	0.35	2.09	0.08
16	1.04	0.25	1.41	0.06
17	0.23	0.09	0.43	0.01
18	0.09	0.04	0.20	0.003
19	0.88	0.22	1.22	0.06
20	0.82	0.20	1.13	0.05
21	0.72	0.18	0.97	0.04
22	0.17	0.06	0.34	0.009
23	0.04	0.02	0.11	0.0003
24	0.65	0.16	0.89	0.04
25	0.45	0.12	0.71	0.02
26	0.06	0.04	0.21	0.002
27	0.61	0.23	0.91	0.00
28	0.31	0.23	0.67	0.00
29	0.28	0.28	0.71	0.00

Nodes A and B are assigned a maximum age (indicate in parentheses) as derived from palynological studies [66], [67]. Letters and numeric codes for each node of the phylogeny of Cistaceae correspond to those shown in Fig. 4. Ma = million years ago; SD = Standard deviation.

## Discussion

An adaptive radiation comprises a group of species that inhabit a variety of environments, differ in morphological and other traits important in utilizing these environments, and are descended from a common ancestor that rapidly speciated over a short period of time [1]. Available phylogenetic and ecological evidence suggests that the *C. salviifolius* lineage of 10 white-flowered species meets the four criteria to strictly test adaptive radiation: common ancestry, rapid speciation, phenotype-environment relationships and trait utility [1], [4].

Monophyly of the white-flowered *Cistus* lineage is strongly supported irrespective of phylogenetic methods and DNA sequences used (Fig. 1). Accordingly, the 12 white-flowered species form a well-defined natural group and fulfil the common ancestry condition.

The concept of rapid speciation is not very well defined, even though a considerable number of species is needed [1]. Asymmetry between sister clades in their number of descendant species is one of the operational standards to distinguish speciation bursts from stochastic background rates [31]. Compared with the two taxa included in the *C. clusii* lineage, the remaining taxa (13) form a sister group (*C. salviifolius* lineage) and can be consider as a significant burst. In fact, asymmetries between both lineages can also be inferred in a temporal pattern. After the split of the most common recent ancestor of the two lineages (1.04±0.25 Ma), a relatively long period of time was necessary to bring about limited (2) extant species in *C. clusii* lineage, in contrast to the 10 species generated in the *C. salviifolius* group (Fig. 4). Alternatively, rapid radiation is also interpreted as high rates of differentiation in comparison to those of flowering plants. The estimated rate of diversification in the *C. salviifolius* lineage was significantly higher (1.46–2.44 species per million years) compared to the median rate of diversification of angiosperm families (0.12 species per million years; with a maximum of 0.39) [32] and to that found in the Andean Valeraniaceae [33], and similar to the explosive radiation described for Andean *Lupinus* [34]. Rapid diversification in the *C. salviifolius* lineage was already predicted by a combination of different sources of evidence prior to performing explicit analysis of radiation: (1) lack of resolution and low support values depicted mainly in the parsimony-based tree because of a low number of polymorphisms [20], which is overcome by increasing the number of DNA substitutions (this paper); (2) short branch lengths and low pairwise sequence divergence (Fig. 4, Table 2); (3) low resolution at the core of the haplotype network [35] (Fig. 3).

In addition to evidence for common ancestry and rapid diversification, the fit of the diverse phenotypes observed in a lineage with their environment is necessary in prediction of adaptive radiations [1]. Our character reconstruction suggests that shifts in leaf features allowing the colonization of different habitats have been related with specific speciation events (Fig. 2). Acquisition of diverse leaf features is associated with recent lineage splits, and thus closely related taxa exhibit different leaf morphologies [26]. Our character state optimization reveals that the most common recent ancestors of four sister species diversified in different environmental conditions (Fig. 2D, 2E, 2F) by means of shifts in leaf shape (Fig. 2A) and leaf pubescence (Fig. 2C). In addition, transition in leaf labdanum secretion is observed in the Bayesian inference (Table 4; Fig. 2B). Trends of correlated evolution between leaf traits and at least one ecological trait (environment, insolation conditions) have been found (Table 5). The barely correlated evolution found in three morphological/ecological traits indicates that shifts in environmental conditions must parallel evolutionary changes in *Cistus* leaf morphology as a whole and not in individual leaf features. Experimental studies testing correlated evolution of all leaf traits should be further performed to analyse compensatory effects (trade-off). Alternatively to sister species approaches, another strong indication of the adaptive value of a trait is when phylogenetically separate, but ecologically similar, species converge or show parallel patterns of variation along similar ecological gradients [36]. Multiple leaf morphological character-states studied across the white-flowered lineage (shape, labdanum secretion, pubescence) have been independently acquired at least twice (Fig. 2).

Evidence that some morphological and/or physiological traits of species are particularly useful is the fourth necessary condition to support the most strict concept of adaptive radiation: trait utility [1]. The adaptive implications of leaf size and shape differences are well documented [37], [38]. In absence of explicit experimental studies (plant translocation, common garden conditions) for all species involved in this adaptive radiation [39], the body of knowledge for particular traits is analysed. Although our six DNA sequence data set rendered certain phylogenetic uncertainty for some sister species relationships because of moderate support (Fig. 1), the most plausible hypothesis allows assessing low character-state reconstruction uncertainty of leaf morphological utility using MacClade optimization and BayesTraits analysis of trait evolution. Leaf size and shape are implicated in important aspects as thermoregulation [40], [41], efficiency of water use [23], [42], photosynthetic potential [43], branching and rooting strategies [44], among others. Moreover, comparative studies have revealed the existence of well-marked ecological and leaf morphological trends [45]. Small leaf size (specifically narrow leaves) are generally favoured under high exposure and/or low water availability as they help to maintain favourable leaf temperature and improves water use efficiency [42], [44]. Indeed, small-leaved species are concentrated at the high exposure end on south-facing slopes in Mediterranean garrigue and Californian chaparral [46]. Although our character reconstruction hypothesis indicates dynamic shifts of leaf shapes, the ancestral state (narrow leaves) appears to have evolved early into linear-lanceolate to elliptic, and then into ovate (plus ovate-lanceolate) leaves independently four times. In fact, helioxerophyllous species (*C. clusii*, *C. munbyi*, *C. libanotis*) show ancestral linear revolute leaves while submesophyllous species with broad, flat leaves inhabit shadier environments (*C. laurifolius*, *C. populifolius*, *C. psilosepalus*) (Table 3, Fig. 2). Leaf shape is not, however, the only phenotypic trait associated with adaptation to dry conditions. Leaf pubescence is reported to be an adaptation to sunnier and hotter environments by reducing transpiration, increasing the probability of water uptake by leaves, maintaining favourable leaf temperature, and protecting against UV-B radiation responsible for photosynthetic inhibition [47], [48]. Accordingly, a combination of leaf trait strategies meets in unrelated species of *Cistus*. Sister species within the white-flowered *Cistus* lineage have different leaf traits related to leaf transpiration. For instance, the ovate leaves of *C. parviflorus* unsuitable for xeric environments are protected by a dense tomentum of stellate hairs. The same is true at a lower extent in *C. salviifolius*. In addition, leaves can be highly reflective in the visible spectrum by covering the upper surface with labdanum, and then decreasing transpiration [49]. The high leaf secretion of resins (labdanum) in the linear-lanceolate leaves of *C. monspeliensis* and *C. ladanifer* may confer a trade-off compared to the narrower leaves of *C. clusii*, *C. mumbyi* and *C. libanotis*, which display linear leaves and lower labdanum concentration (Fig. 2). Further studies are needed to pinpoint whether combination of multiple leaf strategies are equally fit in dry, Mediterranean habitats suffering from dry hot summers and high solar radiation.

In summary, the evolutionary history of the 10 species (13 taxa) of the *C. salviifolius* lineage fits into utilization of the niche space in a novel manner far after the Mediterranean climate establishment [50]. A Mediterranean *Cistus* ancestor with linear, medium labdanum content and glabrous or subglabrous leaves may have spawned new lines of evolution exploiting six pre-existing Mediterranean habitats. Multiple leaf strategies were successfully essayed in the course of speciation to occupy particular environments and become part of the dominant element in the Mediterranean scrub. As far as we know, this is the first documented plant group involved in an adaptive radiation process in the Mediterranean region.

## Materials and Methods

### Sample strategy and DNA sequencing

A total of 36 individuals representing the 21 species of *Cistus*, one of *Fumana*, eight of *Halimium*, one of *Helianthemum* and one of *Tuberaria* was sequenced for four plastid (*trnL-trnF* spacer, *trnS*-*trnG* spacer, *trnK*-*matK* spacer, *rbcL* exon) and two nuclear (*ITS*, ncp*GS*) DNA regions (Table 1; Table S1) to perform phylogenetic analyses and estimate divergence times of *Cistus* and related lineages. In addition, a data set comprising only the white-flowered *Cistus* species (plus *C. parviflorus*) was used to infer character evolution, correlated evolution and haplotype analyses.

Standard primers were used for amplification of the ITS region [51 for 17SE], [52 for ITS4], the *trnL*(UAA)-*trnF*(GAA) [53], the *trnK-matK* [trnK-3914F and matK-1470R, 54] and the *trnS* (GCU)-*trnG* (UCC) [55] spacers. The *rbcL* exon was amplified in two overlapping segments using the following primer combination: 1F-724R and 636F-1460R [56]. A portion of the glutamine synthetase (ncp*GS*) was amplified for the first time in 11 *Cistus* species with the universal primers Gscp687f and Gscp856r [57]. To ensure a homogeneous amplification reaction we design two 24-nucleotide-long primers specific for amplifying and sequencing *Cistus* species (CIS-687f: 5′GTAGCTGGAATCAACATCAGTGG3′ , CIS-856r: 5′GCTTGTTCAGTGATTCTCTGTCAG3′).

After 1–3 min pretreatment at 94°C, PCR conditions for amplification were: 24–39 cycles of 1 min at 94°C, 30 s-1 min at 48–50–55°C and 1–4 min at 72°C (for details see 19). PCR primers were used for cycle sequencing of the spacers, the *rbcL* exon and the ncp*GS* gene while the ITS 5 and ITS 4 [52] primers were used for cycle sequencing the ITS region. Additionally, due to mononucleotide repeat stretches (poly-T, poly-A) the internal primer trnSGpolyTf (5′TTAGATTCTATTTACATTCT3′) was used to sequence the *trnS-trnG* spacer in the purple-flowered species. Sequenced data were assembled and edited using the program Seqed (Applied Biosystems, California). The limits of the regions were determined by position of flanking primers. IUPAC symbols were used to represent nucleotide ambiguities.

### Molecular analyses

#### Phylogenetic analyses

Maximum Parsimony (MP) and Bayesian Inference (BI) analyses were performed on a combined molecular data set of *trnL-trnF*, *trnS*-*trnG*, *trnK*-*matK*, *rbcL*, *ITS* and ncp*GS* sequences. Sequences were aligned using Clustal X 1.62b [58], with further adjustments by visual inspection. All parsimony analyses were conducted using Fitch parsimony [59] with equal weighting of all characters and of transitions/transversions. Heuristic searches were replicated 1000 times with random taxon–addition sequences, tree–bisection–reconnection (TBR) branch swapping, the options MulTrees and Steepest Descent in effect and holding 10 trees per replicate. Internal support was assessed using 5,000,000 bootstrap (BS) replicates [fast stepwise-addition, 60].

To determine the simplest model of sequence evolution that best fits the sequence data, the Hierarchical Likelihood Ratio Test (hLRT) and Akaike Information Criterion (AIC) were implemented using MrModeltest 1.1b [61], [62] in each data set. A Bayesian Inference analysis (BI) was conducted in MrBayes 3.0b4 [63] using two identical searches with two million generations each (four MCMC, chain temperature = 0.2; sample frequency = 100). In both runs probabilities converged at the same stable value after generation 100,000 approximately. A 50% majority-rule consensus tree was calculated using the sumt command to yield the final Bayesian estimate of phylogeny. We used posterior probability (PP) as an estimate of robustness.

#### Molecular dating and diversification rates

Divergence dates were estimated for nodes of the Bayesian consensus tree. To check the constancy of substitution rates we used the Langley and Fitch (LF) test [64]. We rejected the null hypothesis of constant rate (χ^2^ = 5204.26; d.f. = 34) and, then, divergence times were estimated using the r8S 1.71 program [65] with a Penalized Likelihood (PL) approach. Penalized Likelihood was implemented with the Truncated Newton (TN) algorithm. Initial results were obtained under the following parameters: cvstart = 0.5; cvinc = 0.5; cvnum = 10 with cross-validation enforced to estimate the rate smoothing parameter (measure of the rate variation and autocorrelation of rates from clade to clade). The rate smoothing with the lowest crossvalidation score was selected and the dating procedure was repeated with the following parameters: collapse; num_time_guesses = 5 and num_restarts = 5. Crossvalidation suggested 10 as the best smooth parameter. Branching order and branch lengths from 100 Bayesian trees sampled every 10,000 generations after stationary were analyzed to obtain means and standard deviations of clade ages [34]. To convert relative divergence times into absolute time units we used two maximum-age fossil constraints. Palynological studies identified *Helianthemum* pollen in Upper Miocene formations (11 Ma) from France [66] and *Tuberaria* pollen in Pliocene formations (5.3 Ma) from Germany [67].

Species diversification rates, assuming an equal rate of random speciation Yule model, were calculated using the formula SR = [(log_e_(N)–log_e_(N_0_)]/T [34], [68], [69], where N is the total number of extant species in the clade of interest, N_0_ is the initial species diversity, usually taken as 1, and T is the inferred age of the clade (million years). Upper and lower standard deviations of age estimates were used in calculations of speciation rates.

#### Character evolution

Patterns of evolution of six key traits (leaf shape, leaf labdanum secretion, leaf pubescence, soil requirements, insolation conditions, habitat) were explored in the white-flowered *Cistus* lineage using the Bayesian consensus tree (calculated using the same parameters as above). Optimizations were performed in MacClade 4.06 [70] assuming Fitch Parsimony, equal weighting of all characters, transitions among all states equally probable and treating characters as unordered. Character states were determined from literature and personal observations. Samples of *Cistus crispus* and *C. heterophyllus* were used as outgrop sequences.

In addition, to account for values of phylogenetic mapping uncertainty, probabilities of ancestral states for the six traits were estimated individually using the BayesMultiState program, contained in the BayesTraits 1.0 package [71], under Montecarlo Markov Chain (MCMC) method and allowing transitions between character states in both directions. To reduce the autocorrelation of successive samples, 1000 trees were drawn from the distribution of 1.9×10^6^ trees, which equates to sampling every 1900th generation of the chains used in the phylogenetic analysis. As suggested in BayesMultiState manual, to reduce some of the uncertainty and arbitrariness of choosing prior in MCMC studies, we used the hyperprior approach, in concrete the reversible-jump (RJ) hyperprior with a gamma prior (mean and variance seeded from uniform distributions on the interval 0 to 10). Preliminary analyses were run to adjust the *ratedev* parameter until the acceptance rates of proposed changes was around 20–40%. Using *ratedev* settings (Table S2), we ran the RJ MCMC analyses for each trait three times independently for 1.0×10^7^ iterations, sampling every hundredth iteration (to produce 90,000 sampled points) and discarding the first 1,000,000 iterations. All runs gave mostly the same results and we report one of them here. We use the “Addnode” command to find the proportion of the likelihood associated with each of the possible states at each node.

#### Testing correlated evolution

We modelled correlated evolution of discrete binary traits (leaf shape/insolation conditions, leaf shape/habit, labdanum secretion/insolation conditions, labdanum secretion/habit, leaf pubescence/insolation conditions, leaf pubescence/habit) on 1000 Bayesian trees using the BayesDiscrete program, contained in the BayesTraits 1.0 package [71] and the same parameters described above. The method compares the statistical likelihood of a model in which two binary traits are allowed to evolve independently on the tree, with a model in which the two traits are allowed to evolve in a correlated fashion. Evidence for correlated evolution arises if the dependent or correlated model shows significantly better fit to the data than the independent model. As the independent and dependent models are estimated by MCMC, their goodness of fit is compared using the log-Bayes Factor test: 2*log[harmonic mean(dependent model)] – log[harmonic mean (independent model)].

We used one sample per species of the white-flowered lineage given the monophyly of all species [72]. As binary traits are required, we coded traits as followed: leaf shape, 0 linear to elliptic, 1 ovate-lanceolate to ovate; labdanum secretion, 0 zero to eight percent, 1 nine to fifteen; upper leaf pubescence, 0 glabre to subglabre, 1 dense tomentum; insolation conditions, 0 helioxerophyllous to subheliophyllous, 1 subsciophyllous to submesophyllous; environment, 0 bushy and scrub vegetation, 1 woodlands.

#### Haplotype data analysis

Sequences of plastid DNA (*trnL-trnF*, *trnK*-*matK trnS-trnG* and *rbcL*) were combined to analyze relationships among the white-flowered *Cistus* (plus *C. parviflorus*) plastid haplotypes. We used the software TCS 1.21 to infer plastid haplotype ancestry [73]. The program implements a statistical parsimony approach using the algorithm described in Templeton *et al*. [74] to construct haplotype networks. The maximum number of differences among haplotypes, as a result of single substitutions, was calculated with 95% confidence limits and treating gaps as missing data.

## Supporting Information

Table S1 GenBank accession numbers.
(0.10 MB DOC)

Table S2 Bayesian inference of trait evolution of successive iterations of the chain (9,000,000) in the white-flowered *Cistus* lineage by reversible jump Markov chain Monte Carlo. Means±confidence intervals (95%) of the log-likelihoods (Lh) and rate coefficients are shown.
(0.07 MB DOC)

Table S3 List of haplotypes found in 16 species and subspecies of the white-flowered *Cistus* lineage. Variable sites of the sequences of four plastid DNA regions (*trnL*-*trnF*, *rbcL*, *trnK*-*matK*, *trnS*-*trnG*) are shown. Nucleotide position for each data set is numbered from the 5′ to the 3′ DNA ends.
(0.20 MB DOC)
