# Supplementary material for: Adaptive Radiation in Mediterranean Cistus (Cistaceae)
Source: PLoS One. 2009 Jul 23;4(7):e6362. doi: 10.1371/journal.pone.0006362 (PMC2719431; doi:10.1371/journal.pone.0006362)
Supplement: Table S1 — GenBank accession numbers. (0.10 MB DOC) [file pone.0006362.s001.doc]

**Table S1.** **GenBank accession numbers.**

| ***Taxon*** | ***trnL-trnF accession no.*** | ***trnK-matK accession no.*** | ***trnS-trnG accession no.*** | ***rbcL***  ***accession no.*** | ***ncpGS accession no.*** | ***ITS***  ***accession no.*** |
| --- | --- | --- | --- | --- | --- | --- |
| *Cistus* L.  *Cistus albanicus* E.F. Warb. ex Heywood | DQ093057 | DQ093010 | FJ228736 | FJ225880 | GQ258818* | DQ092964 |
| *Cistus albidus* L | DQ093021 | DQ092974 | FJ228730 | FJ492042 | GQ258797* | DQ092932 |
| *Cistus chinamadensis* Bañares et Romero | DQ093033 | DQ092986 | GQ281688* | FJ225866 | GQ258803* | DQ092942 |
| *Cistus clusii* Dunal subsp. *clusii* | DQ093056 | DQ093009 | FJ228739 | FJ225879 | GQ258817* | DQ092963 |
| *Cistus clusii* Dunal subsp. *multiflorus* Demoly | GQ281667* | GQ281700* | GQ281693* | GQ281672* | - | - |
| *Cistus creticus* L. | DQ093025 | DQ092978 | GQ281684* | FJ225862 | GQ258799* | DQ092936 |
| *Cistus crispus* L. | DQ093060 | DQ093013 | GQ281694* | FJ225882 | GQ258820* | DQ093060 |
| *Cistus heterophyllus* Desf. | DQ093036 | DQ092989 | GQ281690* | FJ225868 | GQ258805* | - |
| *Cistus horrens* Demoly | FJ492018 | FJ225848 | GQ281689* | FJ492045 | GQ258804* | GQ281665* |
| *Cistus ladanifer* L. subsp. *africanus* | DQ093048 | DQ093001 | FJ189400 | FJ225874 | GQ258810* | DQ092956 |
| *Cistus ladanifer* L. subsp. *ladanifer* | DQ093043 | DQ092996 | FJ189421 | FJ225872 | GQ258808* | DQ092951 |
| *Cistus ladanifer* L. subsp. *sulcatus* | DQ093046 | DQ092999 | FJ189448 | FJ225873 | GQ258810* | DQ092954 |
| *Cistus laurifolius* L. | DQ093052 | DQ093005 | FJ228731 | FJ225876 | GQ258814* | DQ092959 |
| *Cistus libanotis* L. | DQ093040 | DQ092993 | FJ228732 | FJ225870 | GQ258811* | DQ092948 |
| *Cistus monspeliensis* L. | DQ093059 | DQ093012 | FJ225849 | FJ225881 | GQ258819* | DQ092966 |
| *Cistus munbyi* Pomel | DQ093053 | DQ093006 | FJ228738 | FJ225877 | GQ258815* | DQ092960 |
| *Cistus ochreatus* C. Sm. ex Buch | DQ093032 | DQ092985 | GQ281687* | FJ492043 | GQ258802* | GQ281664* |
| *Cistus osbeckiifolius* Webb ex Christ | FJ492017 | GQ281699* | GQ281685* | FJ492044 | GQ258800* | GQ281663* |
| *Cistus parviflorus* Lam. | DQ093023 | DQ092976 | GQ281683* | FJ225861 | GQ258798* | DQ092934 |
| *Cistus populifolius* L. subsp. *major* (Dunal) Heywood | DQ093049 | DQ093002 | GQ281692* | FJ225875 | GQ258813* | DQ092957 |
| *Cistus populifolius* L. subsp. *populifolius* | DQ093050 | DQ093003 | - | GQ281671* | GQ258812* | - |
| *Cistus pouzolzii* Delile | DQ093054 | DQ093007 | FJ228734 | FJ492046 | GQ258816* | DQ092961 |
| *Cistus psilosepalus* Sweet | DQ093041 | DQ092994 | FJ228737 | FJ225871 | GQ258807* | DQ092949 |
| *Cistus salviifolius* L. | DQ093037 | DQ092990 | GQ281691* | FJ225869 | GQ258806* | DQ092945 |
| *Cistus symphytifolius* Lam*.* | DQ093030 | DQ092983 | GQ281686* | FJ225877 | GQ258801* | DQ092940 |
| *Fumana* (Dunal) Spach  *Fumana thymifolia* (L.) Spach ex Webb | DQ093015 | DQ092968 | - | FJ225850 | - | DQ092926 |
| *Halimium* (Dunal) Spach  *Halimium atlanticum* Humbert & Maire  *Halimium atriplicifolium* (Lam.) Spach  *Halimium calycinum* (L.) K. Koch  *Halimium halimifolium* (L.) Willk. *halimifolium*  *Halimium lasianthum (Lam.) Spach lasianthum*  *Halimium lasiocalicynum* (Boiss. & Reut.) Gross ex Engl. subsp. *riphaeum* (Pau & Font Quer) Maire  *Halmium ocymoides* (Lam.) Willk.  *Halimium umbellatum* (L.) Spach | FJ492006  GQ281666*  DQ093020  FJ492015  GQ281668*  FJ492013  FJ492011  DQ093014 | GQ281695*  FJ225847  DQ092973  GQ281696*  GQ281698*  GQ281697*  FJ225846  DQ092972 | GQ281675*  GQ281680*  GQ281677*  GQ281678*  GQ281682*  GQ281681*  GQ281679*  GQ281676* | FJ492032  GQ281669*  FJ492039  FJ492040  GQ281670*  FJ492037  FJ492035  FJ225857 | GQ258795*  -  GQ258796*  -  -  -  -  - | -  -  DQ092931  GQ281662*  -  GQ281661*  -  DQ092930 |
| *Helianthemum* Mill.  *Helianthemum squamatum* (L.) Dum. Cours. | DQ093016 | DQ092969 | GQ281673* | FJ225851 | GQ258794* | DQ092927 |
| *Tuberaria* Dunal  *Tuberaria guttata* (L.) Fourr. | DQ093018 | DQ092971 | GQ281674* | FJ225853 | - | DQ092929 |

Asterisks (*) after GenBank accession numbers refer to sequences employed for the first time.
