# Supplementary material for: Adaptive Radiation in Mediterranean Cistus (Cistaceae)
Source: PLoS One. 2009 Jul 23;4(7):e6362. doi: 10.1371/journal.pone.0006362 (PMC2719431; doi:10.1371/journal.pone.0006362)
Supplement: Table S2 — Bayesian inference of trait evolution of successive iterations of the chain (9,000,000) in the white-flowered Cistus lineage by reversible jump Markov chain Monte Carlo. Means±confidence intervals (95%) of the log-likelihoods (Lh) and rate coefficients are shown. (0.07 MB DOC) [file pone.0006362.s002.doc]

**Table S2.** Bayesian inference of trait evolution of successive iterations of the chain (9,000,000) in the white-flowered *Cistus* lineage by reversible jump Markov chain Monte Carlo. Means ± confidence intervals (95%) of the log-likelihoods (Lh) and rate coefficients are shown.

| **Trait** | **Ratedev** | **Log-Likelihood (Lh)** | **qAB** | **qAC** | **qAD** | **qAE** | **qAF** | **qAG** | **qBA** | **qBC** | **qBD** | **qBE** | **qBF** | **qBG** | **qCA** | **qCB** | **qCD** | **qCE** |
| --- | --- | --- | --- | --- | --- | --- | --- | --- | --- | --- | --- | --- | --- | --- | --- | --- | --- | --- |
| Leaf shape | 90 | -14.62 ± 0.00 | 39.04 ± 0.19 | 31.08 ± 0.20 | 34.06 ± 0.20 | - | - | - | 35.66 ± 0.19 | 49.08 ± 0.16 | 44.54 ± 0.17 | - | - | - | 38.19 ± 0.20 | 40.96 ± 0.21 | 37.90 ± 0.20 | - |
| Labdanum secretion | 120 | -11.46 ± 0.01 | 39.34 ± 0.23 | 44.69 ± 0.23 | - | **-** | **-** | **-** | 45.74 ± 0.23 | 48.30 ± 0.23 | - | - | - | - | 48.91 ± 0.23 | 46.43 ± 0.23 | - | **-** |
| Leaf pubescence | 130 | -13.18 ± 0.01 | 60.89 ± 0.20 | 62.37 ± 0.19 | - | - | - | - | 42.76 ± 0.24 | 37.79 ± 0.24 | - | - | - | - | 44.09 ± 0.25 | 44.18 ± 0.25 | - | - |
| Soil | 110 | -8.45 ± 0.00 | 33.70 ± 0.14 | 26.83 ± 0.15 | - | - | - | - | 24.22 ± 0.17 | 28.10 ± 0.17 | - | - | - | - | 24.12 ± 0.17 | 32.26 ± 0.17 | - | - |
| Insolation conditions | 100 | -12.55 ± 0.01 | 35.55 ± 0.19 | 33.81 ± 0.19 | 33.96 ± 0.19 | - | - | - | 31.43 ± 0.19 | 34.59 ± 0.19 | 43.55 ± 0.19 | - | - | - | 34.20 ± 0.20 | 36.69 ± 0.20 | 35.38 ± 0.20 | - |
| Environment | 100 | -19.03 ± 0.06 | 45.13 ± 0.23 | 45.89 ± 0.23 | 44.40 ± 0.23 | 44.92 ± 0.23 | 44.60 ± 0.22 | 44.43 ± 0.22 | 44.22 ± 0.23 | 47.19 ± 0.22 | 44.89 ± 0.23 | 46.88 ± 0.22 | 44.36 ± 0.23 | 46.13 ± 0.22 | 41.50 ± 0.22 | 46.10 ± 0.21 | 42.84 ± 0.22 | 45.21 ± 0.22 |

| **Trait** | **qCF** | **qCG** | **qDA** | **qDB** | **qDC** | **qDE** | **qDF** | **qDG** | **qEA** | **qEB** | **qEC** | **qED** | **qEF** | **qEG** | **qFA** | **qFB** | **qFC** | **qFD** |
| --- | --- | --- | --- | --- | --- | --- | --- | --- | --- | --- | --- | --- | --- | --- | --- | --- | --- | --- |
| Leaf shape | - | - | 33.96 ± 0.20 | 37.56 ± 0.20 | 33.52 ± 0.20 | - | - | - | - | - | - | - | - | - | - | - | - | - |
| Labdanum secretion | **-** | **-** | **-** | **-** | **-** | **-** | **-** | **-** | **-** | **-** | **-** | **-** | **-** | **-** | **-** | **-** | **-** | **-** |
| Leaf pubescence | - | - | - | - | - | - | - | - | - | - | - | - | - | - | - | - | - | - |
| Soil | - | - | - | - | - | - | - | - | - | - | - | - | - | - | - | - | - | - |
| Insolation conditions | - | - | 21.86 ± 0.17 | 39.73 ± 0.19 | 24.31 ± 0.17 | - | - | - | - | - | - | - | - | - | - | - | - | - |
| Environment | 42.33 ± 0.22 | 43.14 ± 0.22 | 46.39 ± 0.23 | 48.07 ± 0.23 | 48.92 ± 0.22 | 48.04 ± 0.23 | 47.30 ± 0.22 | 46.50 ± 0.22 | 44.92 ± 0.23 | 47.82 ± 0.22 | 47.81 ± 0.22 | 45.61 ± 0.22 | 45.63  ± 0.22 | 45.63 ± 0.23 | 46.36 ± 0.23 | 47.64 ± 0.23 | 48.23 ± 0.23 | 46.79 ± 0.22 |

| **Trait** | **qFE** | **qFG** | **qGA** | **qGB** | **qGC** | **qGD** | **qGE** | **qGF** |
| --- | --- | --- | --- | --- | --- | --- | --- | --- |
| Leaf shape | - | - | - | - | - | - | - | - |
| Labdanum secretion | **-** | **-** | **-** | **-** | **-** | **-** | **-** | **-** |
| Leaf pubescence | - | - | - | - | - | - | - | - |
| Soil | - | - | - | - | - | - | - | - |
| Insolation conditions | - | - | - | - | - | - | - | - |
| Environment | 47.31 ± 0.23 | 46.18 ± 0.23 | 46.39 ± 0.23 | 49.50 ± 0.22 | 48.75 ± 0.23 | 46.66 ± 0.22 | 47.99 ± 0.22 | 46.76 ± 0.22 |
