# Supplementary material for: Adaptive Radiation in Mediterranean Cistus (Cistaceae)
Source: PLoS One. 2009 Jul 23;4(7):e6362. doi: 10.1371/journal.pone.0006362 (PMC2719431; doi:10.1371/journal.pone.0006362)
Supplement: Table S3 — List of haplotypes found in 16 species and subspecies of the white-flowered Cistus lineage. Variable sites of the sequences of four plastid DNA regions (trnL-trnF, rbcL, trnK-matK, trnS-trnG) are shown. Nucleotide position for each data set is numbered from the 5′ to the 3′ DNA ends. (0.20 MB DOC) [file pone.0006362.s003.doc]

**Table S3.** List of haplotypes found in 16 species and subspecies of the white-flowered *Cistus* lineage. Variable sites of the sequences of four plastid DNA regions (*trnL-trnF, rbcL, trnK-matK, trnS-trnG*) are shown. Nucleotide position for each data set is numbered from the 5’ to the 3’ DNA ends.

| **Nucleotide position** | ***trnL-trnF*** | | | | | | | | | | | | | | | | | | | | | |  |
| --- | --- | --- | --- | --- | --- | --- | --- | --- | --- | --- | --- | --- | --- | --- | --- | --- | --- | --- | --- | --- | --- | --- | --- |
|  | **7** | **8** | **29** | **82** | **116** | **148** | **163** | **177** | **223** | **263** | **270** | **288** | **323** | **359** | **363** | **365** | **366** | **367** | **368** | **380** | **413** | **422** | |
| **Haplotype** |  |  |  |  |  |  |  |  |  |  |  |  |  |  |  |  |  |  |  |  |  |  | |
| *C. albanicus* | C | A | T | G | A | A | C | G | A | C | A | T | - | T | - | - | - | - | - | - | G | G | |
| *C. ladanifer africanus* | C | A | T | G | A | G | C | G | A | C | T | T | - | T | - | - | - | - | - | - | G | G | |
| *C. ladanifer ladanifer & sulcatus* | C | A | T | G | A | G | C | G | A | C | T | T | - | T | - | - | - | - | - | - | G | G | |
| *C. laurifolius* | C | A | T | G | A | G | C | C | A | C | A | T | A | A | A | A | T | T | T | T | T | G | |
| *C. libanotis* | C | A | T | G | A | G | C | G | A | C | A | T | - | - | - | - | - | - | - | T | G | G | |
| *C. monspeliensis* | C | A | T | G | G | G | T | C | C | A | A | T | T | T | T | T | T | A | A | G | G | C | |
| *C. parviflorus* | T | A | T | A | A | G | C | C | A | C | A | G | T | A | A | A | T | T | T | A | T | G | |
| *C. populifolius major* | C | A | T | G | A | G | C | C | A | C | A | T | T | T | A | A | A | T | T | T | G | C | |
| *C. populifolius populifolius* | C | A | T | G | A | G | C | C | A | C | A | T | T | T | A | A | A | T | T | T | G | C | |
| *C. pouzolzii* | C | C | T | G | A | G | C | C | A | C | A | T | T | T | A | A | A | T | T | - | G | G | |
| *C. psilosepalus* | C | A | T | G | A | G | C | C | C | C | A | T | T | A | A | A | T | T | T | T | T | G | |
| *C. salviifolius* | T | A | C | G | A | G | C | G | A | C | T | T | - | T | - | - | - | - | - | - | G | G | |

| **Nucleotide position** | ***rbcL*** | | | | | | | | | | | | |
| --- | --- | --- | --- | --- | --- | --- | --- | --- | --- | --- | --- | --- | --- |
|  | **174** | **376** | **378** | **621** | **717** | **795** | **916** | **934** | **1046** | **1069** | **1297** | **1305** | **1320** |
| **Haplotype** |  |  |  |  |  |  |  |  |  |  |  |  |  |
| *C. albanicus* | C | A | C | C | G | T | C | G | T | C | C | C | G |
| *C. ladanifer africanus* | T | G | C | T | G | C | C | A | A | C | C | A | G |
| *C. ladanifer ladanifer & sulcatus* | T | G | C | T | G | C | C | A | A | C | C | A | G |
| *C. laurifolius* | C | A | C | C | A | T | C | G | T | C | C | C | G |
| *C. libanotis* | T | A | C | T | G | C | C | A | A | C | C | C | G |
| *C. monspeliensis* | C | A | C | T | G | T | C | G | T | C | C | C | G |
| *C. parviflorus* | C | A | T | C | G | T | T | G | T | C | C | C | A |
| *C. populifolius major* | C | A | C | ? | G | T | C | G | T | C | C | C | G |
| *C. populifolius populifolius* | C | A | C | T | G | T | C | G | T | C | C | C | G |
| *C. pouzolzii* | C | A | C | T | G | T | C | G | T | C | C | C | G |
| *C. psilosepalus* | C | A | C | C | G | T | C | G | T | T | C | C | G |
| *C. salviifolius* | T | A | C | T | G | C | C | A | A | C | A | C | G |

**Table S3 *(****Continued)*

| **Nucleotide position** | ***trnK-matK*** | | | | | | | | | | | | | | |
| --- | --- | --- | --- | --- | --- | --- | --- | --- | --- | --- | --- | --- | --- | --- | --- |
|  | **33** | **105** | **109** | **113** | **131** | **297** | **302** | **317** | **344** | **402** | **428** | **434** | **458** | **595** | **607** |
| **Haplotype** |  |  |  |  |  |  |  |  |  |  |  |  |  |  |  |
| *C. albanicus* | G | C | G | A | T | G | T | T | C | C | G | G | G | G | G |
| *C. ladanifer africanus* | G | C | G | A | T | G | A | T | C | C | G | C | G | G | G |
| *C. ladanifer ladanifer & sulcatus* | G | C | G | A | T | G | A | T | C | C | G | C | G | G | G |
| *C. laurifolius* | G | A | G | A | C | G | T | T | T | C | T | G | T | G | T |
| *C. libanotis* | G | C | T | A | T | G | T | T | C | C | G | G | G | G | G |
| *C. monspeliensis* | G | A | G | A | T | G | T | T | C | C | G | G | G | G | G |
| *C. parviflorus* | G | C | T | A | T | G | T | T | C | A | G | G | G | G | G |
| *C. populifolius major* | G | A | G | C | T | G | T | T | C | C | G | G | G | G | G |
| *C. populifolius populifolius* | G | A | G | C | T | G | T | T | C | C | G | G | G | G | G |
| *C. pouzolzii* | G | A | G | A | T | G | T | T | C | C | G | G | G | G | G |
| *C. psilosepalus* | T | A | G | A | T | G | T | T | C | C | G | G | G | G | G |
| *C. salviifolius* | G | C | G | A | T | T | T | G | C | C | G | G | G | T | G |

| **Nucleotide position** | ***trnK-matK*** | | | | | | | | | | | | | |
| --- | --- | --- | --- | --- | --- | --- | --- | --- | --- | --- | --- | --- | --- | --- |
|  | **853** | **869** | **871** | **889** | **918** | **947** | **1012** | **1031** | **1089** | **1126** | **1159** | **1316** | **1323** | **1334** |
| **Haplotype** |  |  |  |  |  |  |  |  |  |  |  |  |  |  |
| *C. albanicus* | G | T | G | C | T | C | G | A | C | A | A | C | C | A |
| *C. ladanifer africanus* | G | G | G | C | T | C | G | A | C | A | A | C | C | C |
| *C. ladanifer ladanifer & sulcatus* | G | G | G | C | T | C | G | A | T | A | A | C | C | C |
| *C. laurifolius* | G | G | G | C | T | C | G | A | C | A | A | C | C | C |
| *C. libanotis* | G | G | G | C | T | C | G | A | C | A | A | C | C | C |
| *C. monspeliensis* | T | G | A | C | T | C | G | A | C | A | A | C | C | C |
| *C. parviflorus* | G | G | G | C | T | C | G | A | C | A | A | C | C | C |
| *C. populifolius major* | G | G | G | C | G | C | G | A | C | A | G | C | C | C |
| *C. populifolius populifolius* | G | G | G | C | G | C | G | A | C | A | G | C | C | C |
| *C. pouzolzii* | G | G | G | C | T | C | T | A | C | A | A | C | C | C |
| *C. psilosepalus* | G | G | G | C | T | T | G | A | C | A | A | A | G | C |
| *C. salviifolius* | G | G | G | A | T | C | G | C | C | C | A | C | C | C |

**Table S3 *(****Continued)*

| **Nucleotide position** | ***trnS-trnG*** | | | | | | | | | | | | | | | | | | | | | | | |
| --- | --- | --- | --- | --- | --- | --- | --- | --- | --- | --- | --- | --- | --- | --- | --- | --- | --- | --- | --- | --- | --- | --- | --- | --- |
|  | **102** | **104** | **105** | **118** | **126** | **155** | **158** | **217** | **227** | **236** | **255** | **303** | **312** | **333** | **342** | **350** | **366** | **367** | **414** | **423** | **436** | **528** | **533** | **569** |
| **Haplotype** |  |  |  |  |  |  |  |  |  |  |  |  |  |  |  |  |  |  |  |  |  |  |  |  |
| *C. albanicus* | T | G | C | T | T | C | T | C | T | T | C | A | T | C | T | T | G | A | C | A | G | T | T | G |
| *C. ladanifer africanus* | T | G | C | T | T | C | T | C | T | T | C | A | T | A | T | T | G | A | A | A | G | C | T | G |
| *C. ladanifer ladanifer & sulcatus* | T | G | C | T | T | C | T | C | T | T | C | A | T | A | T | G | G | A | A | C | G | C | T | G |
| *C. laurifolius* | T | G | C | T | G | C | T | C | T | T | C | A | T | A | T | T | G | A | C | A | G | C | T | G |
| *C. libanotis* | T | G | C | T | T | A | T | C | T | T | T | A | T | A | T | T | G | A | C | A | T | C | T | A |
| *C. monspeliensis* | T | G | C | C | T | C | T | C | T | T | C | A | G | A | T | T | T | T | C | A | G | C | A | G |
| *C. parviflorus* | T | G | C | T | T | C | T | C | T | T | C | A | G | C | T | T | G | A | C | A | G | T | T | G |
| *C. populifolius major* | G | G | C | T | T | C | G | C | T | T | C | T | T | A | T | T | G | A | C | A | G | C | T | G |
| *C. populifolius populifolius* | G | G | C | T | T | C | G | C | T | T | C | A | T | A | T | T | G | A | C | A | G | C | T | G |
| *C. pouzolzii* | T | G | C | T | T | C | T | C | T | T | C | A | T | A | T | T | T | A | C | A | G | C | T | ? |
| *C. psilosepalus* | T | G | C | T | T | C | T | C | T | G | C | A | T | A | T | T | G | A | C | A | G | C | T | G |
| *C. salviifolius* | T | T | A | T | T | C | T | A | C | T | C | A | T | A | G | T | T | C | C | A | G | C | T | G |
